# Oxygen Enemata

**Published:** 1887-12

**Authors:** J. H. Kellogg

**Affiliations:** Battle Creek, Mich.


### OXYGEN ENEMATA.

By J. H. Kellogg, M. D., Battle Creek, Mich.

Some weeks ago, while employing the carbon-dioxide and sulphuretted hydrogen enemata, it occurred to me that oxygen gas might be employed advantageously in the same manner. Having at that time under treatment a case of lithiasis, I resolved to make a trial of the administration of oxygen by enema, in the hope that by the introduction of oxygen directly into the portal circulation the conversion of uric acid into urea might be aided, and my patient thereby relieved. I was at first deterred from the attempt by the supposition that oxygen would probably give pain, as it has been claimed that the pain sometimes occasioned in the employment of sulphuretted hydrogen by the Bergeon method is produced by the accidental injection of small quantities of atmospheric air. I determined, however, to try the experiment, and made the first injection of two litres of pure oxygen gas on June 20th. At this time the patient, a man of twenty-eight years, was passing very great quantities of uric acid daily, a large quantity of crystals being thrown down from every specimen of urine passed. The symptom had persisted most stubbornly for weeks, in spite of the enforcement of a non-nitrogenous diet, the patient also taking, by direction, from three to five pints of hot water daily. The beneficial effect of either regimen or treatment was so slight as to be scarcely perceptible. Two litres of oxygen were administered regularly each day, about 10 A. M. At the end of three days the excess of uric acid disappeared entirely from the urine, and has appeared only twice since, and then in small quantity, when the injection had been discontinued for a day or two. At the beginning of treatment the patient's skin was very muddy, sclerotics dingy, tongue coated, and he complained of a constant and very annoying brassy taste in the mouth, and distressing headache. The brassy taste and headache both disappeared very soon after the injections were begun, and there has been a steady improvement in every other particular. The coat has disappeared from the tongue, the skin and sclerotics have become nearly normal in appearance, and the patient has gained several pounds in weight, though before he had been constantly losing weight for some weeks, and seems to be in every respect improved. The patient, a very intelligent gentleman, attributes his improvement almost wholly to the employment of the oxygen, as the measures which had been previously employed had had little or no favorable influence upon his condition, and no change in regimen or treatment has been made with the exception of the administration of the oxygen. It seems to the writer that there may be a large field of usefulness for oxygen employed after this method. The advantages of the employment of oxygen by enema, as compared with the inhalation of oxygen, are very great.

1. In the inhalation of oxygen, only a very small portion of the gas inhaled can be used, the greater part being wasted; as experiment has shown that not more than one-fourth of the oxygen contained in the air is extracted by the lungs during the passage of the air through them in ordinary respiration, and that a very much smaller proportion is absorbed when the quantity of air passing through the lungs is increased, as in deep breathing. Hence, in order to get into the system any considerable quantity of oxygen, it is necessary for a person to inhale a very large amount, making the treatment very expensive.

2. Experiments which have been made for the purpose of testing the therapeutic value of oxygen inhalations have shown that the proportion of oxygen absorbed by the lungs is not proportionally increased by the increase of the percentage of oxygen in the respired air. Indeed, the reported results of careful experiments show that very little more oxygen was actually received by the system when breathing pure oxygen than in the breathing of ordinary atmospheric air. When oxygen is introduced into the alimentary canal the entire quantity introduced is absorbed, and thus utilized, making it possible to introduce a known quantity of oxygen into the system, and in a manner which leaves no room for doubt that it has been received and appropriated.

3. In cases in which it may be considered desirable that a considerable quantity of oxygen should be introduced into the system for the purpose of encouraging the oxidation processes by which effete matters are disintegrated and prepared for alimentation, especially those which are acted upon by the liver, the oxygen enema offers a means by which the gas can be applied where it is most needed. Oxygen received by the lungs passes at once to the left side of the heart, and thence through the arterial system to the entire body, so that the liver receives no more than its due proportion of the gas. Consequently, a very considerable increase in the quantity of oxygen absorbed through the lungs would scarcely increase to an appreciable degree the amount of oxygen available for use by the liver. This fact is made particularly conspicuous when we reflect that the chief source of blood-supply to the liver is the portal circulation. It is apparent, however, that when oxygen is introduced into the lower part of the alimentary canal, it is at once received through the venous absorbents into the portal circulation, through which it is conveyed directly to the liver, precisely where it is needed, thus making the entire amount of oxygen administered available for the purpose for which it is administered. Is it not, then, apparent that this method of administering oxygen possesses, for cases of the sort referred to, an immense advantage over its use by inhalation?

4. The process of digestion is, from a chemical standpoint, chiefly a process of hydration and oxidation. The experiments of Dujardin-Beaumetz, performed some time ago, showed that the drinking of oxygenated water exerts a very beneficial effect upon the digestive process in persons suffering with slow digestion. (I have not seen any extended account of experiments of Dujardin-Beaumetz; I have simply seen in medical literature a mention of the fact stated.) May it not be true that, in some cases, at least, the difficulty under which the digestive organs labor is a lack of a sufficient supply of oxygen in the blood, or, at any rate, that the introduction of oxygen into the portal circulation by oxygen enemata may be found to facilitate the digestive process? It is very easy to make a practical test of the matter, and I am now engaged in a series of experiments in the use of oxygen in various forms of dyspepsia, the results of which I shall report at some future time.

5. It seems to me that oxygen enemata may be employed advantageously in a great variety of cases; in fact, in all cases in which there is such a disturbance of the normal interchange of gases in the lungs as deprives the system of its proper amount of oxygen. The mucous membrane of the intestines presents an absorbing surface; very small, it is true, when compared with the amount of surface presented to the air in the lungs, and yet it is sufficiently great to allow of the introduction into the system of a large amount of oxygen in addition to that which can be gotten in through the lungs; and this additional quantity, though small when compared with the total amount received by the lungs, may be of sufficient value to the system to be of immense advantage to it, especially on account of its introduction at this particular point in the circulation. The notable functional disturbances of the stomach which accompany various pulmonary disorders, such as emphysema, chronic bronchial catarrh, chronic pleurisy, pneumothorax, etc., suggest a very important relation between the digestive function and the quantity of oxygen received through the lungs. The same relation is also suggested by the frequency with which dyspepsia occurs among sedentary persons, who are habitually air-starved in consequence of deficient muscular activity. Oxygen enemata may prove a valuable remedy in cases of this sort, as well as in others in which an additional supply of oxygen is needed.

I have carefully questioned patients respecting the retention of the oxygen injected, and have found that it has always been retained without difficulty, and does not pass off as flatus from the bowels or eructations from the stomach. This fact offers good evidence of its absorption; but to determine the fact beyond the possibility of question, I made, on July 20, an experiment on a guinea-pig. After placing the animal under chloroform, the abdomen was opened, and the intestines drawn out and spread out in such a way that the dark portal veins were in full view. A quantity of gas was then injected into the rectum, and, to my great satisfaction, I found that the dark venous blood assumed a bright red hue, almost equal to that of arterial blood, within less than one minute after the injection of the gas, showing the rapidity with which the absorption of the oxygen takes place. To confirm the result, I allowed the oxygen to escape from the bowels, afterwards replacing it, and repeating the experiment several times. In each instance the color of the blood in the mesenteric veins assumed its ordinary dark purple color immediately after the oxygen was withdrawn, while the bright color returned almost instantly when the new supply of oxygen was introduced. I have been informed by Dr. Hal Wyman, of Detroit, that he made a similar experiment upon dogs a number of years ago, and that he observed the same result as regards the effect of the oxygen on the color of the blood in the portal circulation, but I understand that he made no attempt to employ oxygen in this manner for therapeutic purposes. Indeed, I am not aware that the gas has been used in this manner prior to my own experiment in the case which I have reported. I shall, however, be much surprised if I learn that I am entitled to the credit of priority in this matter, as it seems to me that I must have been very dull not to have sooner thought of this mode of employing so valuable an agent as oxygen.

It has been long known to physiologists that in certain fishes the mucous lining of the alimentary canal performs a very important part of the work of the respiratory system. Some members of the gar family are killed as quickly by cutting off the supply of oxygen to the alimentary canal as by interrupting the gill respiration. Great numbers of illustrations might be given from the lower classes of the animal kingdom in which the entire process of respiration is carried on by the mucous lining of the alimentary canal. Why, then, should not man, whose alimentary canal is very much more extensive and better fitted for absorption than in lower animals, also be able to receive a very appreciable and efficient amount of oxygen through this channel? In my experiments thus far I have found that patients take, without inconvenience, from one to two litresof the gas at a time, and this dose can be repeated three or four times in the twenty-four hours without any difficulty. I have never found any serious discomfort to be produced, although in large quantities it does not seem to be tolerated quite so readily as the carbon-dioxide and sulphuretted hydrogen mixture.

I am employing the oxygen enemata in a variety of cases, in one of which, a case of phthisis, the beneficial results arising from the gas have been surprising. When the treatment was begun the patient had for some time had an evening temperature varying from two to three degrees above normal. The morning temperature was from 99° to 99⅗°. Within forty-eight hours after the use of the oxygen was begun the temperature fell to normal, and did not rise above 99° at any time, until about two weeks after the beginning of treatment, when the febrile symptoms reappeared as a result of an operation for the relief of a troublesome hæmorrhoid, following which the patient, by imprudence, contracted a slight cold.

In the use of the oxygen enema I have employed a new form of apparatus, which I devised some time ago, and which I described in a paper read before the late meeting of the Medical Association in Chicago. The especial advantages of this apparatus are convenience in use, precision in dose, and an easily-regulated and perfectly uniform flow—*Therapeutic Gazette.*

